# Predicting current and future areas of ecological suitability for *Lutzomyia longipalpis* sensu lato in the Americas

**DOI:** 10.1093/jme/tjaf184

**Published:** 2025-12-19

**Authors:** Sydney DeWinter, Grace K Nichol, Christopher Fernandez Prada, Amy L Greer, J Scott Weese, Katie M Clow

**Affiliations:** Department of Population Medicine, Ontario Veterinary College, University of Guelph, Guelph, ON, Canada; Department of Population Medicine, Ontario Veterinary College, University of Guelph, Guelph, ON, Canada; Department of Pathology and Microbiology, Faculty of Veterinary Medicine, University of Montreal, Saint-Hyacinthe, QC, Canada; Department of Biology, Trent University, Peterborough, ON, Canada; Department of Pathobiology, Ontario Veterinary College, University of Guelph, Guelph, ON, Canada; Department of Population Medicine, Ontario Veterinary College, University of Guelph, Guelph, ON, Canada

**Keywords:** sand flies, ecological suitability, leishmaniasis, climate change, *Lu. longipalpis* sensu lato

## Abstract

*Leishmania infantum* is one of the etiologic agents of leishmaniases in mammals. In the Americas, numerous sand fly species within the *Lutzomyia* genus drive *Leishmania* spp. transmission, such as the species complex *Lutzomyia longipalpis* sensu lato. It remains unknown if climatic changes could facilitate range expansion of *Lu. longipalpis*, creating conditions for local transmission in previously non-endemic regions. The objectives of this study were to identify the climatic and environmental variables of importance for *Lu. longipalpis*, current ecologically suitable area across the Americas, and determine future areas of ecological suitability under 30-year time periods. Occurrence records were obtained from GBIF, WRBU, and literature searches. Historic climate data (1981–2010) and projection data for Shared Socioeconomic Pathway 3-7.0 for time periods 2041–2070, and 2071–2100 were obtained from CHELSA, along with topographic data from EarthEnv. Using MaxEnt species distribution modelling algorithms, data were incorporated to identify areas which currently are or may become suitable for *Lu. longipalpis*. Ecological variables such as terrain ruggedness index, number of growing degree days at which mean daily air temperature is above >10 °C, Köppen–Geiger climate classification, and mean daily air temperature of the coldest quarter, were identified as drivers of suitability. Current regions of ecological suitability include areas from the southern United States to northern Argentina. Suitability may expand northward and increase within its current range, specifically in parts of Mexico and Brazil. Findings from this study identify climate and environmental variables impacting *Lu. longipalpis* distribution, and regions of potential range expansion.

## Introduction

*Leishmania* spp. are zoonotic, protozoal parasites causing disease in animals (namely dogs), and humans (Davies et al. 2000, Kaszak et al. 2015, Von Stebut 2015, Araujo and Gondim 2020, Wagner et al. 2020). In dogs, infection with *Leishmania infantum* causes canine leishmaniosis, which is a multisystemic disease that ranges from subclinical to severe and fatal disease (Saridomichelakis 2009, Kaszak et al. 2015, Von Stebut 2015, Morales-Yuste et al. 2022). Dogs also play a significant role in *L. infantum* transmission, as they are the reservoir of the pathogen. In humans, *L. infantum* causes visceral leishmaniasis. If left untreated, visceral leishmaniasis is often fatal. Globally, approximately 12 million people and 2.5 million dogs suffer from some form of leishmaniasis (Kaszak et al. 2015, Von Stebut 2015, World Health Organization 2023).

In South America, the lifecycle and subsequent transmission of *L. infantum* is dependent on the presence of *Lutzomyia* spp., which are small, phlebotomine sand flies of the order Diptera (de Almeida et al. 2013, Maroli et al. 2013, Carvalho et al. 2015, Cecílio et al. 2022). One of the primary vectors is the species complex *Lutzomyia longipalpis* sensu lato (herein in referred to as *Lu. longipalpis*) (Soares and Turco 2003, Bauzer et al. 2007, Souza et al. 2017, Sousa-Paula et al. 2021). As part of their lifecycle, adult female *Lu. longipalpis* take blood meals from mammals where they can ingest *L. infantum* amastigotes and transmit the parasite to another host during a subsequent bloodmeal (Saridomichelakis 2009, Harhay et al. 2011, Maroli et al. 2013, Kaszak et al. 2015, Oliveira et al. 2018). *Lutzomyia longipalpis* is found in countries throughout South and Central America, including Argentina, Bolivia, Brazil, Colombia, Costa Rica, El Salvador, Guatemala, Honduras, Mexico, Nicaragua, Paraguay, and Venezuela (Young and Duran 1994, Quintana et al. 2013, Peterson et al. 2017, Santos et al. 2018). They thrive in warm, humid habitats with abundant vegetation and refugia (González et al. 2006, González et al. 2010, Ocampo et al. 2012, Martín et al. 2020, Avila-Jimenez et al. 2021, Senne et al. 2021, Cecílio et al. 2022, DeWinter et al. 2024).

In the United States and Canada, there is no endemic vector-borne transmission of *L. infantum* via *Lu. longipalpis* due to the absence of this species. Other *Lutzomyia* spp. are present in the areas of the United States but their role in *L. infantum* transmission is still under investigation, and likely much less significant than *Lu. longipalpis* (Lawyer and Young 1987, Lawyer et al. 1987, Comer et al. 1994, Schaut et al. 2015). That being said, dogs are regularly imported from countries with endemic transmission of *L. infantum*, some of which are infected, providing repeat introduction events (Anderson et al. 2016, Wagner et al. 2020, Bouattour et al. 2021, Julien et al. 2021, Gradoni et al. 2022, Blackmore et al. 2023). Risk already exists for non-vector-borne transmission from infected dogs via breeding or reproduction. Large outbreaks of canine leishmaniosis in the United States, including an outbreak in a New York foxhound kennel in 1999, and cases across 18 states and two Canadian provinces from 2000 to 2003 were linked to breeding practices (Gaskin et al. 2002, Travi et al. 2002, Duprey et al. 2006, Schaut et al. 2015). Additionally, with repeat introduction events, if *Lu. longipalpis* underwent range expansion, this could facilitate vector-borne transmission of *L. infantum* and pose a significant threat to veterinary and public health.

Ongoing climatic changes have created suitable conditions in more northern areas for several other arthropod vectors and facilitated range expansion (e.g., *Ixodes scapularis, Aedes albopictus*) (Clow et al. 2017, Kamal et al. 2018, Khan et al. 2020, Alkishe et al. 2021). However, it remains unknown if these changes could lead to expanded ecological suitability specifically for *Lu. longipalpis*, and thus, facilitate range expansion of this species into more northern areas.

Ecological niche models (ENM), also known as species distribution models, are utilized to estimate the relationship between species occurrence records and the climatic and habitat characteristics of the regions where they are found (Phillips et al. 2006, González et al. 2010, Elith et al. 2011, Escobar 2020). These models are often utilized to forecast potential areas of suitability under differing climate change scenarios (Phillips et al. 2006, González et al. 2010, Elith et al. 2011). Among the available niche modelling tools, the most widely utilized is the maximum entropy (MaxEnt) approach (Morales et al. 2017). Construction of ecological niche models, using presence-only species data, can determine both current and future areas that are ecologically suitable for *Lu. longipalpis.* These models assist in identifying regions which may not be known to be suitable, and thus, contribute to risk assessment of *Leishmania* spp. transmission in current non-endemic regions. Further, understanding regions which can become suitable in the future can provide insight into potential regions of expansion, if dispersal mechanisms are present.

The objectives of this study were to (1) determine the current ecologically suitable areas across South America and the southern reaches of North America for *Lu. longipalpis*, (2) identify climatic and environmental factors impacting ecological suitability of *Lu. longipalpis*, and (3) determine the future ecologically suitable areas under two different 30-year periods.

## Methods

### Study Area

Terrestrial regions of South, Central and North America were selected for this study. For current and projected ecological suitability, the area extended from the southern United States to the whole of South America. Specifics regarding the spatial extent of the study area are available in the Supplementary Material (Zurell et al. 2020) (Supplementary Table S1).

### Data Acquisition

Species presence-only data for *Lu. longipalpis* were obtained from the Global Biodiversity Information Facility (GBIF; https://www.gbif.org), the Disease Vectors Database (now discontinued, obtained directly from researchers), the Walter Reed Biosystematics Unit (WRBU; https://wrbu.si.edu), and from extensive searches of the literature (Ward et al. 1983, Navin et al. 1985, Ward et al. 1985, Young et al. 1987, Morrison et al. 1993, Herrero et al. 1994, França-Silva et al. 2005, Salomón and Orellano 2005, de Fátima Freire de Melo Ximenes et al. 2006, De Oliveira et al. 2006, De Resende et al. 2006, Beatriz et al. 2008, de Oliveira et al. 2008, Filho and Brazil 2009, Souza et al. 2009, Amóra et al. 2010, Fernández et al. 2010, Salomón et al. 2011, de Souza et al. 2012). To be included, records needed to be observed by a human (e.g., evidence of occurrence taken from field notes or literature) or machine (e.g., a photograph or video, etc,) between the years of 1981 and 2010. Preserved species (ie, specimens maintained in museums or universities collections) were not eligible for inclusion. For each specimen, the longitude, latitude, and year of collection was ascertained.

Presence points were projected using QGIS Version 3.22.1 (https://qgis.org/en/site, 2024). All duplicate presence points and points that were less than 5 km apart were removed to reduce artificial clustering and correct for spatial biases within the samples (Slatculescu et al. 2020). Remaining presence points were imported into MaxEnt species distribution modelling software (Phillips et al. 2025, Version 3.4.4).

For initial model consideration, bioclimatic and topographic variables were selected based on previous literature regarding the ecology of *Lu. longipalpis* (Table 1) (Soares and Turco 2003, González et al. 2006, González et al. 2010, Ocampo et al. 2012, Martín et al. 2020, Avila-Jimenez et al. 2021, Senne et al. 2021, Cecílio et al. 2022, Elias et al. 2022, DeWinter et al. 2024). Therefore, fourteen bioclimatic variables and one habitat variable (e.g., the Köppen–Geiger climate classification), for the time-period of 1981–2010, were obtained from the Climatologies at High Resolution for the Earth Land Surface (i.e., CHELSA) database (https://chelsa-climate.org) (Peel et al. 2007, Karger et al. 2017, Karger et al. 2018, Brun et al. 2022a, 2022b). Five additional topographic variables, including topographic position index, terrain ruggedness index, and elevation median, minimum, and maximum, were obtained from EarthEnv (https://www.earthenv.org) (Amatulli et al. 2018). All data were downloaded to 30 arc-sec (∼1 km) resolution, available in coordinate reference system WGS 84 (CRS84). Due to the low dispersal potential (typically < 100 m but can disperse ∼ beyond 500 m in some instances) of *Lu. longipalpis*, it is expected that this resolution is appropriate (Morrison et al. 1993, De Oliveira et al. 2013, Galvis-Ovallos et al. 2018).

**Table 1.** Summary of all bioclimatic and topographical variables investigated in *Lu. longipalpis* s.l ecological niche model construction

Variable	Name and unit of measurement	Included in final model?
**Bio 1**	Mean annual air temperature (°C)	No
**Bio 5**	Mean daily maximum air temperature of the warmest month (°C)	Yes
**Bio 6**	Mean daily minimum air temperature of the coldest month (°C)	No
**Bio 9**	Mean daily air temperatures of the driest quarter (°C)	No
**Bio 11**	Mean daily air temperatures of the coldest quarter (°C)	Yes
**Bio 12**	Annual precipitation amount (kg m^−2 ^year^−1^)	No
**Bio 14**	Precipitation amount of the driest month (kg m^−2 ^month^−1^)	No
**Bio 17**	Mean monthly precipitation amount of the driest quarter (kg m^-2 ^month^−1^)	Yes
**Gdd5**	Growing degree days heat sum above 5 °C (°C)	No
**Gdd10**	Growing degree days heat sum above 10 °C (°C)	No
**Gsl**	Growing season length TREELIM	Yes
**Gst**	Mean temperature of the growing season TREELIM (°C)	No
**Ngd5**	Number of growing degree days at which mean daily air temperature > 5 °C (°C)	No
**Ngd10**	Number of growing degree days at which mean daily air temperature > 10 °C (°C)	Yes
**Kg2**	Köppen–Geiger climate classification	Yes
**Elevation_Median**	Median elevation (masl)	No
**Elevation_Min**	Minimum elevation (masl)	No
**Elevation_Max**	Maximum elevation (masl)	Yes
**Tpi**	Topographic position index	Yes
**Tri**	Terrain ruggedness index	Yes

Projected climate data were also obtained from CHELSA. The climate projection Coupled Model Intercomparison Project 6 (CMIP6) ISIMIP3 was selected. Specifically, the General Circulation Model (GCM) GFDL-ESM4, from the National Oceanic and Atmospheric Administration, Geophysical Fluid Dynamics Laboratory, Princeton, USA (NOAA GFDL) was selected. Determined by ISMIP, this GCM is of the highest priority, and is recommended to be used when not all projection GCMs are being utilized (Karger et al. 2017). Shared Socioeconomic Pathways (SSPs) outline emission scenarios driven by socioeconomic assumptions for future time periods, ranging from 'sustainability' (SSP 1-2.6), to 'fossil fueled development' (SSP 5-8.5). For this study, SSP 3-7.0 was selected as 'middle of the road' SSPs are typically considered to be more stable than their extreme counterparts. Shared Socioeconomic Pathway 3-7.0 assumes that CO_2_ emissions would cause temperatures to rise by 3.6 °C by 2100. For this SSP, two different 30-year time periods were selected; 2041–2070 and 2071–2100 (Riahi et al. 2017).

### Data Preparation

All presence data and ecological data (current and future) were imported into QGIS Version 3.22.1 to be geoprocessed to the same resolution of 30 arc-seconds and coordinate reference system (WGS 84 [CRS84]).

To reduce collinearity in the data, a correlation matrix was created. Bioclimatic, habitat, and topographical data around each presence point was extracted from QGIS and imported into RStudio (Version 4.4.3). Highly correlated variables were recorded (>0.80), but all variables (regardless of correlation) were included in the initial model iteration. Following stepwise removal of variables with a permutation importance of 0%, any highly correlated variables remaining were removed based on which had the lowest permutation importance. No highly correlated variables were included in the final model (Table 1).

### Current Ecological Suitability Model

Models were created using presence-only MaxEnt modelling algorithms. This algorithm automatically infers the unknown distribution, *Q*(*x*), from known distributions. Specifically, this assumes *Lu. longipalpis* is equally likely to occur anywhere on the landscape. The initial model, based on historic data (1981–2010), was constructed by importing presence data and rasterized ecological data into MaxEnt (Version 3.4.4). A *k-*fold cross-validation was selected, where *k *= 4 (Merow et al. 2013, Radosavljevic and Anderson 2014, Morales et al. 2017, Phillips et al. 2017, Slatculescu et al. 2020). Cross-validation randomly partitions occurrence data into equal-sized groups (“folds”), creating models from these folds (Phillips et al. 2017). This method is ideal for small datasets, as all data are used for validation (ie, used for training and test data) (Phillips et al. 2017). Selected feature classes included linear, quadratic, product, and threshold with a clog-log output (Table 2).

**Table 2.** MaxEnt model parameter settings utilized in ecological niche model construction, along with ecological variables included in final model investigating ecological suitability for *Lu. longipalpis* s.l in North and South America

	Parameter setting
**Feature class(es)**	Linear, quadratic, product, and threshold
**Output**	Clog log
**Regularization multiplier**	1.0
**Replicated run type**	*k*-fold cross validation
***k***	4
**Final variables**	BIO 5, BIO 11, BIO 17, KG2, GSL, NGD10, TPI, TRI, Elevation_Max

Models were built in an iterative process, with permutation importance of each variable informing inclusion into the next model iteration. If a variable had a permutation importance of >0%, then, it was retained in the next iteration of the model. The final model was reached when all variables in the model had a permutation importance of >0%. Following the identification of the final model, the regularization multiplier was changed from 0.5, 1 (default), 1.5, and 2.0. (Merow et al. 2013, Radosavljevic and Anderson 2014, Morales et al. 2017, Phillips et al. 2017, Slatculescu et al. 2020). For each change in the regularization multiplier, the area under the receiver operating curve (AUC), and mean test omission rate of the minimum training presence were used to identify which multiplier was indicative of superior model fit.

To further understand variable trends and impact, independent response curves and a jackknife test of regularized training gain were generated in an output MaxEnt (Version 3.4.4) file.

### Future Ecological Suitability Models

The ecological variables deemed to have an impact on *Lu. longipalpis*, based on the final current model (i.e., the variables forming their current ecological niche), were carried forward in the future projection models.

### Model Evaluation

The mean area under the receiver operating characteristic curve (AUC) for training and test data, the test omission rate of the minimum training presence, and the maximum training sensitivity plus specificity (MTSS) were used to assess fit (Radosavljevic and Anderson 2014, Slatculescu et al. 2020). The AUC provides a threshold-independent assessment of overall model parameters, whereas the omission rate of the minimum training presence indicates the least-suitable ecological conditions (Radosavljevic and Anderson 2014, Slatculescu et al. 2020). Models returning an AUC closest to 1.0, and omission rate closest to the predicted value of 0 are indicative of superior fit (Fielding and Bell 1997, Radosavljevic and Anderson 2014, Slatculescu et al. 2020). Additional information, reported according to the Overview, Data, Model, Assessment, and Prediction (i.e., ODMAP) protocol, are available in the Supplementary Material (Table S1).

## Results

### Vector Species Records

**Fig. 1.**
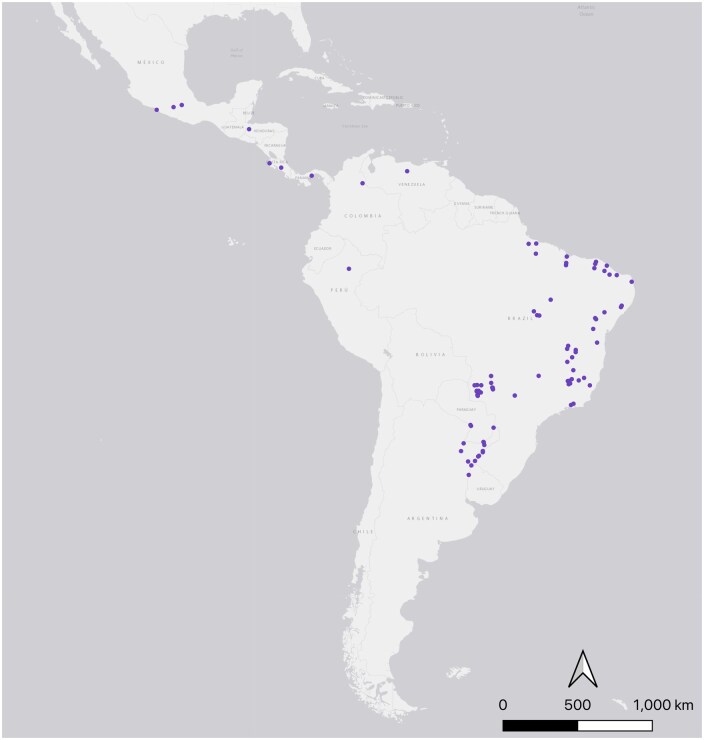
The geographic location of presence points for *Lu. longipalpis* s.l (*n* = 101). Presence points were identified on the basis of human or machine observation, from 1981 to 2010. Map made in QGIS (Version 3.22.1).

A total of 110 *Lu. longipalpis* presence records were obtained. Following de-duplication and rarefaction, 101 presence points were eligible for inclusion (Fig. 1).

### Current (1981–2010) Ecological Niche Model

Following the removal of ecological variables with a permutation importance of 0%, and highly correlated variables, the final model predicting the ecological suitability of *Lu. longipalpis* at ∼1 km resolution from 1981-2010 included mean daily maximum air temperature of the warmest month (°C; Bio 5), mean daily air temperatures of the coldest quarter (°C; Bio 11), mean monthly precipitation amount of the driest quarter (kg m^−2 ^month^−1^; Bio 17), the Köppen–Geiger climate classification (Kg2), growing season length according to TREELIM (Paulsen and Körner 2014) (Gsl), number of growing degree days at which mean daily air temperature > 10 °C (Ngd10), topographic position index (Tpi), terrain ruggedness index (Tri), and maximum elevation (masl) (Elevation_Max) (Peel et al. 2007, Karger et al. 2017, Amatulli et al. 2018, Karger et al. 2018, Brun et al. 2022a, 2022b) (Table 2).

The model had an area under the curve (AUC) of the test data of 95.3%, with a standard deviation of 0.009%. This AUC is indicative of a good model fit (Fielding and Bell 1997). The mean omission rate of the test data, based on the minimum training presence was 0.063. The mean maximum test sensitivity plus specificity (MTSS) was 0.042. Terrain ruggedness index, number of growing degree days at which mean daily air temperature > 10 °C (°C), and the Köppen–Geiger climate classification were found to have the greatest impact on ecological suitability for *Lu. longipalpis* from 1981 to 2010 (51.0%, 24.4%, and 7.8%, respectively; Table 3).

**Table 3.** MaxEnt outputs for the final current ecological niche model, based on presence data collected from 1981 to 2010^a^

Variable	Permutation importance (%)
**Mean daily maximum air temperature of the warmest month (Bio 5)**	1.0
**Mean daily air temperatures of the coldest quarter (Bio 11)**	7.4
**Mean monthly precipitation amount of the driest quarter (Bio 17)**	2.1
**Growing season length TREELIM**	0.3
**Köppen**–**Geiger climate classification**	7.8
**Maximum elevation**	5.1
**Topographic position index**	0.8
**Terrain ruggedness index**	51.0
**Number of growing degree days at which mean air temperature > 10 °C**	24.4

a The current ecological niche model had an AUC = 95.3% ± 0.009, mean test omission rate (minimum training presence) = 0.063, and mean maximum test sensitivity plus specificity (MTSS) = 0.042.

**Fig. 2.**
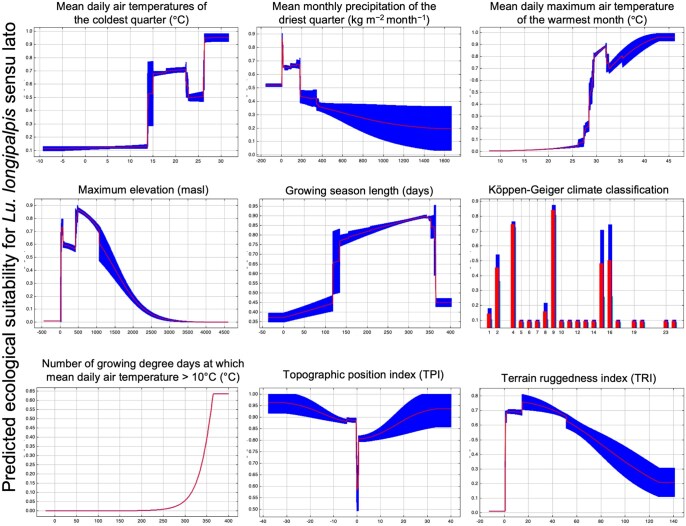
Independent response curves depicting the dependence of predicted current ecological suitability for *Lu. longipalpis* s.l on each modelled ecological variable (in isolation). Red bands indicate the mean response, and blue bands indicate the standard deviation of the variable. Graphs were made in MaxEnt (3.4.4) (Phillips et al. 2025).

Based on the independent response curves generated with this model, ecological suitability for *Lu. longipalpis* increases when the mean daily air temperature of the coldest quarter exceeds 15 °C, mean daily maximum air temperature of the warmest month exceeds 25 °C, number of growing degree days at which mean daily air temperature > 10 °C, and growing season length increases. Likewise, ecological suitability decreases when the mean monthly precipitation of the driest quarter, maximum elevation, terrain ruggedness index increase. Based on Köppen–Geiger climate types, tropical savanna climate or tropical wet and dry climate (classification Aw), humid subtropical climate/warm temperate climate (classification Cfa), humid subtropical climate—monsoon influenced (Cwa), and oceanic/subtropical highland climate (Cwb) were all associated with relatively high (>0.70) suitability for *Lu. longipalpis* (Fig. 2).

**Fig. 3.**
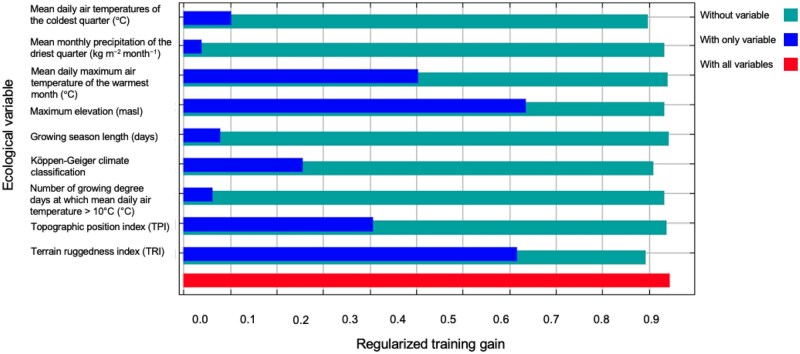
Jackknife test of variable importance of *Lu. longipalpis* s.l current ecological niche model. The jackknife test determines theregularized training gain of each variable in the final model, demonstrating which variables have the greatest impact on model gain when in isolation (blue), or when omitted from the model (teal). Figure was made using MaxEnt (3.4.4) (Phillips et al. 2025).

Results from the jackknife test of variable importance indicate that maximum elevation, followed (masl), followed by terrain ruggedness index, lead to the greatest gain in the model when examined in isolation. Further, omission of these variables decrease model gain the most (Fig. 3).

**Fig. 4.**
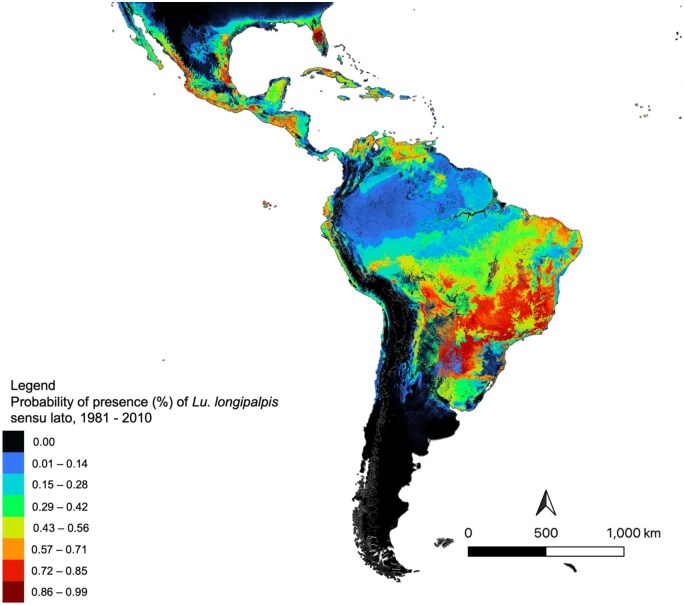
The current ecological suitability for *Lu. longipalpis* s.l, based on historic bioclimatic data from 1981 to 2010. Warmer colours are indicative of highly suitable areas, whereas cooler colours indicate areas with low suitability for *Lu. longipalpis* s.l. Map was created in MaxEnt (Version 3.4.4) and exported into QGIS (Version 3.22.1).

Using presence and bioclimatic data from 1981 to 2010, current ecological suitability for *Lu. longipalpis* spanned from the southern United States (e.g., Texas and Florida) to northern Argentina. Specifically, these ecologically suitable regions included southern Florida (United States), Cuba, the Bahamas, Jamaica, Haiti, and the Dominican Republic, eastern and southern regions of Mexico, Belize, western Guatemala, El Salvador, and Nicaragua, Honduras, northern Colombia and Venezuela, some regions of western Ecuador, and northwestern Peru, Guyana, throughout Brazil (specifically eastern regions, and much of southern Brazil), Paraguay, Bolivia, and northern regions of Argentina (Fig. 4).

#### Projected Future Ecological Suitability Models, SSP 3-7.0

Projections into 2041–2070, and 2071–2100 were applied to the current ecological niche of *Lu. longipalpis*, based on presence data collected between 1981 to 2010.

**Fig. 5.**
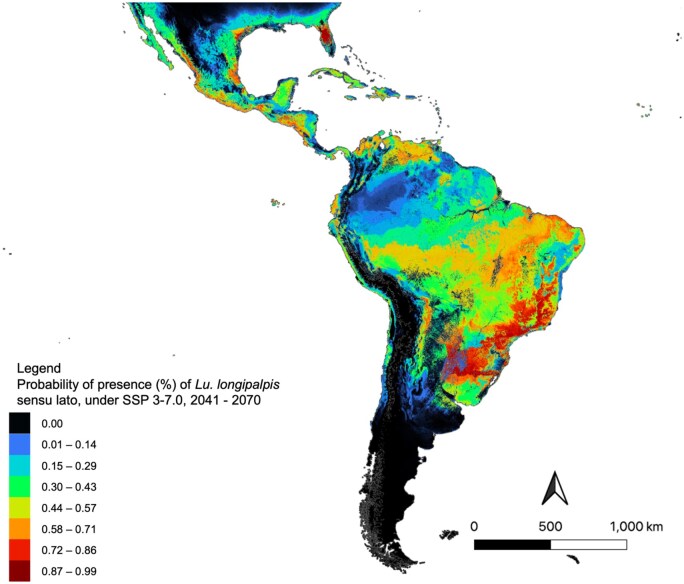
Future ecological suitability for *Lu. longipalpis* s.l, using projected bioclimatic data for Shared Socioeconomic Pathway 3-7.0, 2041–2070. Warmer colours are indicative of highly suitable areas, whereas cooler colours indicate areas with low suitability for *Lu. longipalpis* s.l. Map was created in MaxEnt (Version 3.4.4) and exported into QGIS (Version 3.22.1).

From 2041 to 2070, the model forecasted some slight decreases in suitability in Cuba, Haiti, and the Dominican Republic. Increases in suitability were forecasted in southern Texas and Florida (United States), northwestern/western Mexico, eastern Mexico, Panama, northern Colombia, throughout Venezuela, western Ecuador and Peru, Bolivia, Uruguay and Paraguay, and northern Argentina. In Brazil, there was widespread increase in suitability. This included central Brazil, along with southern Brazil (Fig. 5).

**Fig. 6.**
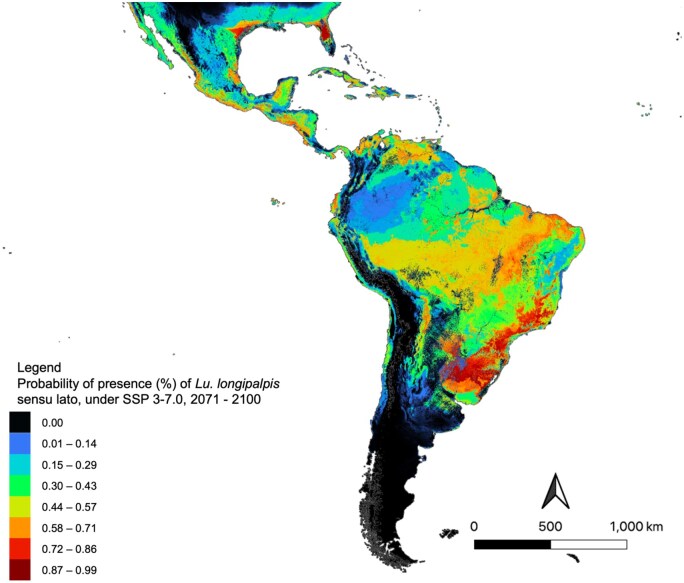
Future ecological suitability for *Lu. longipalpis* s.l, using projected bioclimatic data for Shared Socioeconomic Pathway 3-7.0, 2071–2100. Warmer colours are indicative of highly suitable areas, whereas cooler colours indicate areas with low suitability for *Lu. longipalpis* s.l. Map was created in MaxEnt (Version 3.4.4) and exported into QGIS (Version 3.22.1).

From 2071 to 2100, forecasted suitability was similar to that of the 2041–2070 map, with a further increase in suitability in Texas and Florida (United States), northern Uruguay, and across central Brazil. In eastern Brazil, suitability slightly reduced in some regions (Fig. 6).

## Discussion

As climatic changes accelerate, the ecological niches of arthropod species are shifting across the globe (Clow et al. 2017, Kamal et al. 2018, Khan et al. 2020, Alkishe et al. 2021). It is therefore critically important for both human and animal health to evaluate what areas can ecologically support vector species now and into the future. In this study, the current and future ecological suitability of the Americas was evaluated for *Lu. longipalpis*, which is a vector of human and animal health significance, due to its ability to transmit *Leishmania* spp. such as *L. infantum* (Kaszak et al. 2015, Von Stebut 2015, Morales-Yuste et al. 2022). Ecological suitability is driven by the terrain ruggedness index, number of growing degree days at which mean air temperature > 10 °C, the Köppen–Geiger climate classification, and mean daily air temperatures of the coldest quarter. Suitability shifts for *Lu. longipalpis*, under SSP 3-7.0, were identified in North America, specifically northern Mexico and southern Texas and Florida (United States), along with central regions of South America, largely in central Brazil.

Topographic variables such as the terrain ruggedness index and maximum elevation, were drivers of ecological suitability in the projection model. Regarding their high permutation importance, both variables had the greatest gain for determining ecological suitability in the model. Terrain ruggedness index is a measurement of terrain changes, based on elevation differences between each cell. A higher index is indicative of a more rugged surface (ArcGIS 2020). From the ecological niche model, the highest suitability for *Lu. longipalpis* was associated with a relatively low terrain ruggedness index of less than 20 m. Once the index increased beyond 140 m, suitability was negligible for *Lu. longipalpis.* An index of less than 80 m is a relatively level terrain surface, while an index above 117 m is considered to be slightly rugged (ArcGIS 2020). Similarly, maximum elevation was also associated with a steep decrease in suitability. Elevations lower than 1000 metres above sea level (masl) were associated with high suitability, whereas elevations exceeding 3000 masl were not suitable for *Lu. longipalpis*. Previous studies have also reported the importance of elevation for *Lutzomyia* spp. (Colla-Jacques et al. 2010, Altamiranda-Saavedra et al. 2020, Avila-Jimenez et al. 2021). Therefore, rugged, elevated terrain is unsuitable for *Lu. longipalpis*, and likely presents a topographic barrier for dispersal.

Degree days are a fundamental component of the ecology of all vectors. They are the number of days and degrees where the temperature is above or below a fixed threshold and used in the estimation of insect growth and development during the growing season (Oshaghi et al. 2009, ArcGIS 2023). A higher number of growing degree days are indicative of longer and warmer growing conditions (ArcGIS 2023). In this study, the number of growing degree days at which mean air temperature was above 10 °C was positively correlated with ecologically suitability for *Lu. longipalpis.* This aligns well with the current knowledge on temperature thresholds for these sand flies. From previous research, it is known that *Lutzomyia* spp. can survive in regions where the temperatures are above 10 °C, and at least 15 °C for a minimum of three months (Von Stebut 2015, Cecílio et al. 2022). The response curves indicate ecological suitability increases as the number of growing degree days at which mean air temperature > 10 °C increases. Specifically, there is a steep increase in ecological suitability for *Lu. longipalpis* once the number of growing degree days exceeds 300 days.

The Köppen–Geiger climate classification also contributed to ecological suitability for *Lu. longipalpis*. This study identified several suitable climate classifications for *Lu. longipalpis*, such as those defined by tropical wet and dry climate, tropical and subtropical climate, and humid subtropical climate. Characteristics of these classifications include regions of high humidity/precipitation, which is well-documented in previous research on *Lutzomyia* spp. (Young and Duran 1994, González et al. 2010, Berrozpe et al. 2017). Moisture levels within the environment are important to prevent dry, arid habitats, and maintain soil moisture (Feliciangeli 2004, González et al. 2010, Berrozpe et al. 2017). Soil moisture is important for the lifecycle of *Lu. longipalpis*, as eggs are laid in organic material and undergo other life stages in this location (Feliciangeli 2004, González et al. 2010, Berrozpe et al. 2017).

Mean daily air temperatures of the coldest quarter was a driver for ecological suitability in both the current (1981–2010) and projected ecological niche models. Previous research has illustrated the importance of temperature on ecological suitability for *Lu. longipalpis* (Young and Duran 1994, Guzman and Tesh 2000, Colla-Jacques et al. 2010, de Almeida et al. 2013, Rivas et al. 2014, Oliveira et al. 2018, Del Carro et al. 2020, Martín et al. 2020, Pereira et al. 2020, DeWinter et al. 2024). *Lutzomyia longipalpis* can survive at temperatures as low as 15 °C but experience reduced activity. Emergence (i.e., development from pupae to adult) increases with temperatures of 20 °C or higher while peak activity and abundance are observed when temperatures range from 25 °C to 30 °C (Guzman and Tesh 2000, Colla-Jacques et al. 2010, de Almeida et al. 2013, Rivas et al. 2014, Oliveira et al. 2018, Del Carro et al. 2020, Martín et al. 2020, Pereira et al. 2020, DeWinter et al. 2024). These previous findings are well-supported in the response curve for mean daily air temperatures of the coldest quarter. From the response curve, suitability for *Lu. longipalpis* steeply increased once the mean daily air temperature exceeded 15 °C, and was highest when temperatures are between 15 °C and ∼22 °C, and over 25 °C. Further, as these temperatures are for the coldest quarter, temperatures that are conducive to high activity would be beneficial for *Lu. longipalpis*, as they greatly impact by temperature fluctuations (Young and Duran 1994, Guzman and Tesh 2000, Colla-Jacques et al. 2010, de Almeida et al. 2013, Rivas et al. 2014, Oliveira et al. 2018, Del Carro et al. 2020, Martín et al. 2020, Pereira et al. 2020, DeWinter et al. 2024). Therefore, low temperatures during the coldest quarter would be considerably limiting for these sand flies.

Currently, *Lu. longipalpis* has been found throughout southern Mexico, southern Nicaragua, northern Costa Rica, southeastern Brazil, northeastern Paraguay, and eastern Bolivia. *Lutzomyia longipalpis* has not been recorded in Belize, Ecuador, Peru, Chile, Guyana, Suriname, or French Guiana (Young and Duran 1994, de Almeida et al. 2013, Maroli et al. 2013, Carvalho et al. 2015, Cecílio et al. 2022). The current ecological niche model inferred larger regions of suitability from the environmental data at each presence point (Phillips et al. 2006, González et al. 2010, Elith et al. 2011, Escobar 2020). Differences between known distribution and ecological niche indicate that while areas currently exist which are ecologically suitable (i.e., small parts of Ecuador and Peru, Cuba), there are likely other factors which impact *Lu. longipalpis* suitability. A mechanism of dispersal into a novel region also needs to be considered. *Lutzomyia longipalpis* have low dispersal potential (i.e., disperse > 100 m from site of emergence) and are sensitive to wind (De Oliveira et al. 2013, Galvis-Ovallos et al. 2018). However, they have been found to enter buses and trains, and thus, could be transported longer distances (Costa and de Miranda-Santos 2011). Other vectors, such as mosquitoes, are capable of long-distance dispersal through ships and airplanes, but this has not yet been found for *Lu. longipalpis* ­(Service 1997).

Based on model projections, *Lu. longipalpis*-suitable regions could expand as far north as southern Texas and Florida (United States), and as far south as north reaches of Argentina. There is expected to be a widespread increase in suitable regions across Brazil. Similar findings were reported in a previous study by Peterson et al.(2017). While *Lu. longipalpis* appears to have a well-defined ecological niche, projections seem to indicate a southward expansion of ecological suitability, and to a lesser extent, a northward one.

These ecological niche models can provide insight for surveillance and prevention efforts. Targeted monitoring can be conducted in the future, specifically in regions determined to be suitable for *Lu. longipalpis*, but with no known presence records. Surveillance could be utilized to validate the findings of this model. Knowledge on which ecological factors drive suitability can be utilized to further refine these monitoring efforts during certain seasons, or during certain weather events. Prevention efforts can be considered in highly suitable areas, including those at the edge of suitability, such as including residual sprays of houses and animal shelters, insecticide-treated mosquito nets, or other chemical repellents (Alexander and Maroli 2003).

Based on the ecological niche models, significant northward expansion of *Lu. longipalpis* into North America is not expected. Therefore, *L. infantum-*transmission in Canada and the United States via these sand flies is unlikely, even if there are infected reservoir hosts in the country (Kaszak et al. 2015, Von Stebut 2015, Baneth and Solano-Gallego 2022, Morales-Yuste et al. 2022).

While the findings presented are valuable, there are limitations which should be acknowledged. First, there was a lack of consistent surveillance data available for *Lu. longipalpis*, and likely bias in the sampling that did occur. Therefore, it is possible there are regions where *Lu. longipalpis* is established but not recorded, and thus, the predicted ecological suitability could be altered if these presence data are in ecological areas not represented by the dataset utilized in this study. Further, *Lu. longipalpis* is not one species, but a species complex (Bauzer et al. 2007, Souza et al. 2017, Sousa-Paula et al. 2021). However, much surveillance data available does not further specify which population was collected. Therefore, it is entirely possible that ecologies may differ, but this was not able to be accounted for in the model. Additionally, while the CHELSA dataset provided many important variables for considering vector ecology, some were still lacking. Specifically, the number of frost-free days were not included in the model. These data were available in other datasets, such as Envirem (https://envirem.github.io), but did not include projection data. Finally, these models can only be used to inform regions of ecological suitability for *Lu. longipalpis*. They cannot be used to identify regions of *Lu. longipalpis*-driven transmission of *Leishmania* spp., as other components of the *Lu. longipalpis* lifecycle, and *Leishmania* spp. transmission cycle were not considered in the models.

## Supplementary Material

tjaf184_Supplementary_Data
