# Supplementary material for: Predicting current and future areas of ecological suitability for Lutzomyia longipalpis sensu lato in the Americas
Source: J Med Entomol. 2025 Dec 19;63(2):tjaf184. doi: 10.1093/jme/tjaf184 (PMC13016989; doi:10.1093/jme/tjaf184)
Supplement: tjaf184_Supplementary_Data [file tjaf184_supplementary_data.docx]

| **ODMAP section** | | | **ODMAP subsection** | | | **ODMAP elements** | | | | |
| --- | --- | --- | --- | --- | --- | --- | --- | --- | --- | --- |
|  | | **O**verview |  | | Authorship |  | | · Authors: S. DeWinter, G.K. Nichol, C. Fernandez-Prada, A.L. Greer, J.S. Weese, K.M. Clow | | |
|  |  |  |  |  |  |  |  | · Contact email: sdewinte@uoguelph.ca | | |
|  |  |  |  |  |  |  |  | · Title: Predicting current and future areas of ecological suitability for *Lutzomyia longipalpis* sensu lato in the Americas | | |
|  |  |  |  |  |  |  |  | · DOI | | |
|  |  |  |  | | Model objective |  | | · SDM objective/purpose: determining the ecological suitability of regions across North and South America for *Lu. longipalpis,* along with identifying environmental variables which impact ecological suitability, and how this may change in the future. | | |
|  |  |  |  |  |  |  |  | Ecological inference / explanation | Mapping / interpolation | Forecast / transfer |
|  |  |  |  |  |  |  |  | · Main target output: e.g., Suitable vs. unsuitable habitat (based on ecological suitability) | | |
|  |  |  |  | | Taxon |  | | *Lu. longipalpis* sensu lato (Lutz & Neiva, 1912) | | |
|  |  |  |  | | Location |  | | · Location of study area: North and South America | | |
|  |  |  |  | | Scale of analysis |  | | · Spatial Extent: (34.4085593112930113) N (-19.5516645190139968) E (-56.2175384619569982) S (-125.2779452040000052) W | | |
|  |  |  |  |  |  |  |  | · Spatial resolution: 30 arc-sec (~1 km) | | |
|  |  |  |  |  |  |  |  | · Temporal extent/time period: 1981 - 2010, projected into 2041 - 2070 and 2071 - 2100 | | |
|  |  |  |  |  |  |  |  | · Type of extent boundary: Natural | | |
|  |  |  |  | | Biodiversity data overview |  | | · Observation type: field survey/human observation | | |
|  |  |  |  |  |  |  |  | · Response/data type: presence-only | | |
|  |  |  |  | | Type of predictors |  | | · Climatic, topographic, habitat | | |
|  |  |  |  | | Conceptual model |  | | Hypotheses about species-environment relationships:  · Ecological variables related to temperature and precipitation/moisture [broadly] are expected to have considerable impact on suitability for *Lu. longipalpis.* Specifically, it is expected that as temperature, degree days, and precipitation/moisture increase, as will suitability. Elevation is expected to be a barrier, with higher elevations reducing suitability.  · Due to climatic changes, it is expected that suitability will increase for *Lu. longipalpis* in the future | | |
|  |  |  |  | | Assumptions |  | | Critical model assumptions:  · Presence-only model is utilized 🡪 therefore, absence is not assumed in the model  · Presence data represent a random sample of space  · Prior Q(x) assuming that *Lu. longipalpis* is equally likely to be anywhere on the landscape [i.e., every pixel has the same probability of being selected as background] | | |
|  |  |  |  | | SDM algorithms |  | | · Model algorithms: Maximum entropy (MaxEnt) modelling algorithms | | |
|  |  |  |  |  |  |  |  | · Justification of model complexity: A range of regularization multipliers (RMs) were used for the model (0.5 – 2.0) to ensure that the model was not over- or underfitted to the data. The AUC, omission rates, and MTSS were compared for each RM | | |
|  |  |  |  |  |  |  |  | · Is model averaging/ensemble modelling used? Yes, *k-*fold cross validation (*k =* 4) was used, with the average between all four models being used | | |
|  |  |  |  | | Model workflow |  | | · Conceptual description of modelling steps including model fitting, assessment and prediction   1. Ecological variables which were presumed to have some impact on suitability for *Lu. longipalpis*, were included, which lead to the inclusion of twenty variables for initial model iteration 2. To reduce collinearity, a correlation matrix was generated including all twenty variables. Highly correlated variables were noted, but no action was taken at this time 3. All fourteen variables were included in the initial model iteration, with variables returning a permutation importance (PI) = 0% being removed, and the model re-ran. This was done until all remaining variables had a PI > 0% 4. Previously recorded highly correlated variables were considered. If highly correlated variables remained, the ones with the lowest PI were removed in a step-wise manner until the final model was reached. The final model included all variables with a PI > 0 with no variables that were highly correlated | | |
|  |  |  |  | | Software, codes and data |  | | · Specify modelling platform incl. version, key packages used: MaxEnt species distribution modelling software, Version 3.4.4 | | |
|  |  |  |  |  |  |  |  |  | | |
|  |  |  |  |  |  |  |  | · Specify availability of data, e.g. data links: CHELSA data are available here (<https://chelsa-climate.org>), and EarthEnv data are available here ((<https://www.earthenv.org>) | | |
|  | | **D**ata |  | | Biodiversity data |  | | Taxon names:  · Domain – Eukaryota  · Kingdom – Animalia  · Phylum – Arthropoda  · Class – Insecta  · Order – Diptera  · Family – Psychodidae  · Genus – *Lutzomyia*  · Species name – *Lutzomyia longipalpis* sensu lato | | |
|  |  |  |  |  |  |  | | · Details on taxonomic reference system: Linnaean | | |
|  |  |  |  |  |  |  | | · Ecological level: Individual | | |
|  |  |  |  |  |  |  | | · Biodiversity data source: accessed in the literature between February - March 2025 | | |
|  |  |  |  |  |  |  | | Sampling design: spatial design (e.g. random, uniform, stratified), temporal design, nestedness  · Variable, as data were obtained from a variety of sources. Recorded on the basis of human or machine observation (i.e., field notes, literature [previous research], or photographs, videos, RSI) | | |
|  |  |  |  |  |  |  | | · Sample size per taxon: 101 | | |
|  |  |  |  |  |  |  | | · Country/region masl, if applicable: variable, ranges from 119 masl – 6575 masl | | |
|  |  |  |  |  |  |  | | Details on scaling, if applicable: e.g., rasterisation of polygon maps, spatial and temporal thinning, measures to address spatial uncertainties  · All ecological data were downloaded at the same resolution of 30 arc-sec and therefore did not need to be re-scaled | | |
|  |  |  |  |  |  |  | | Details on data cleaning/filtering steps, if applicable: e.g., taxonomically, spatially, temporally, outlier presence/treatment  · Only *Lu. longipalpis* were included in the model [presence data]  · Data were rarefied to prevent any artificial clustering or spatial biases in the data | | |
|  |  |  |  |  |  |  | | · Details on absence data collection, if applicable: N/A | | |
|  |  |  |  |  |  |  | | Details on background data derivation, if applicable: e.g., spatial and temporal extent, spatial and temporal buffer, bias correction (e.g. target group sampling)  · Spatial extent: (34.4085593112930113) N (-19.5516645190139968) E (-56.2175384619569982) S (-125.2779452040000052) W  · Temporal extent: 1981 – 2010  · Q(x) 🡪 assumes *Lu. longipalpis* is equally likely to be anywhere on the landscape  · Used cross validation to generate background points (more information below) | | |
|  |  |  |  |  |  |  | | Details on potential errors and biases in data, if applicable: e.g., detection probability, misidentification potential, geo-referencing errors, sampling bias  · Potential for sampling bias – it is possible that *Lu. longipalpis* populations exist in other regions, but have not been observed/recorded 🡪 presence points are derived from data available in the literature | | |
|  |  |  |  |  | Data partitioning |  |  | · Selection of training data (for model fitting): *k-*fold cross validation 🡪 data are split into *k* (in this case, *k =* 4) subsets (folds), and models are created leaving out each subset/fold. This method uses all data for validation and is good for smaller datasets | | |
|  |  |  |  |  |  |  |  | · Selection of validation data (withheld from model fitting, used for estimating prediction error for model selection, model averaging or ensemble): *k-*fold cross-validation | | |
|  |  |  |  |  |  |  | | · Selection of test (truly independent) data: derived from *k-*fold cross-validation | | |
|  |  |  |  | | Predictor variables |  | | · State predictor variables used: bio1,5,6,9,11,12,14,17, gdd5, gdd10, gsl, gst, ngd5, ngd10, kg2, elevation-mean, elevation_median, elevation_min, elevation_max, tpi, tri | | |
|  |  |  |  |  |  |  | | · Details on data sources: e.g., URL/DOI, accession date, database version: CHELSA (Version 2.1) data are available here (<https://chelsa-climate.org>), and EarthEnv data are available here ((<https://www.earthenv.org>) | | |
|  |  |  |  |  |  |  | | Spatial resolution and spatial extent of raw data, if different from biodiversity data  · Resolution: 30 arc-sec (~1km)  · Spatial Extent: (83.9998611111) N, (179.9998611111) E, (-90.0001388888) S, (-180.0001388888) W *data were clipped to the extent referenced above* | | |
|  |  |  |  |  |  |  | | · Map projection (coordinate reference system): WGS 84 (CRS84) | | |
|  |  |  |  |  |  |  | | · Temporal resolution and temporal extent of raw data, if applicable: 1981 – 2010 for current, 2041 – 2070 and 2071 – 2100 for the projection data | | |
|  |  |  |  |  |  |  | | · Details on data processing and on spatial, temporal and thematic scaling: e.g. upscaling/downscaling, transformations, normalisations, thematic aggregations (e.g. of land cover classes), measures to address spatial uncertainties: data were downloaded as .tiff files 🡪 spatial data were then clipped by masking to fit the previously indicated extent before being reprojected to .asc files | | |
|  |  |  |  |  |  |  | | · Details on measurements errors and bias, when known: N/A | | |
|  |  |  |  |  |  |  | | · Details on dimension reduction of variable set, if applicable – if model-based, this should be contained in **M**odel section (element: Details on pre-selection of variables) | | |
|  |  |  |  | | Transfer data for projection |  | | · Details on data sources: e.g., URL/DOI, accession date, database version: CHELSA (Version 2.1) data are available here (<https://chelsa-climate.org>), and EarthEnv data are available here ((<https://www.earthenv.org>) | | |
|  |  |  |  |  |  |  | | · Spatial extent: (83.9998611111) N, (179.9998611111) E, (-90.0001388888) S, (-180.0001388888) W | | |
|  |  |  |  |  |  |  |  | · Spatial resolution: 30 arc-sec (~1km) | | |
|  |  |  |  |  |  |  |  | · Temporal extent/time period: 2041 – 2070 and 2071 - 2100 | | |
|  |  |  |  |  |  |  |  |  | | |
|  |  |  |  |  |  |  | | · Models and scenarios used: CMIP6 ISIMIP3, GFDL-ESM4 (National Oceanic and Atmospheric Administration, Geophysical Fluid Dynamics Laboratory), SSP 3-7.0 [priority 1] | | |
|  |  |  |  |  |  |  | | Details on data processing and scaling (see above):  · Prior to downloading, bias in the data were corrected using trend-preserving bias correction  · Data were downloaded as .tiff files 🡪 spatial data were then clipped by masking to fit the previously indicated extent before being reprojected to .asc files | | |
|  |  |  |  |  |  |  | | · Quantification of novel environmental conditions and novel environmental combinations: e.g., distance to training data | | |
|  | | **M**odel |  | | Variable pre-selection |  | | · Details on pre-selection of variables, if applicable: initial variables were included based on their suspected/hypothesized significance on determining ecological suitability for *Lu. longipalpis.* This initially included 14 variables. All these variables were included in the initial iteration of the model, and removed based on their permutation importance (variables with a PI = 0 were removed and model was re-run) | | |
|  |  |  |  | | Multicollinearity |  | | Methods for identifying and dealing with multicollinearity (Dormann, et al. 2013) or justification if multicollinearity is not explicitly dealt with  · Assessed through a correlation matrix 🡪 >0.80 was considered to be highly correlated  · Highly correlated variables were noted but not removed from model to ensure that variables impacting *Lu. longipalpis* were not removed  · Model was built in a step-wise manner, where any variable with a PI = 0 was removed and the model was re-run until all only variables with a PI > 0 were retained in the model  · Next, previously recorded highly correlated variables were considered 🡪 if highly correlated variables remained, the ones with the lowest PI were removed in a step-wise manner until the final model was reached | | |
|  |  |  |  | | Model settings |  | | Models settings for all selected algorithms (including default settings of specific platforms/packages, weighting of data etc.)  · Feature class(es): linear, quadratic, product, threshold  · Output: cloglog  · Regularization multiplier: 1.0  · Replicated run type: *k*-fold cross validation  · *k:* 4 | | |
|  |  |  |  |  |  |  | | · Details on relevant model settings for extrapolation beyond sample range, if applicable: Clamping | | |
|  |  |  |  | | Model estimates |  | | · Assessment of model coefficients: N/A | | |
|  |  |  |  |  |  |  | | · Assessment of variable importance: permutation importance (%), independent response curves, and jackknife test of variable importance | | |
|  |  |  |  | | Model selection / Model averaging / Ensembles |  | | · Model selection strategy: changed the RM for model, from 0.5 – 2.0, and assessed individual models based on AUC, omission rates, and MTSS. RM = 1.0 was chosen as this yielded the highest AUC and lowest omission rate among the generated models. | | |
|  |  |  |  | | Non-independence correction/analyses |  | | · Method for addressing spatial autocorrelation in residuals: rarefication of data points to ensure independence, repeat observations not used | | |
|  |  |  |  |  |  |  | | · Method to account for nested data: N/A | | |
|  |  |  |  |  | Threshold selection |  |  | · Details on threshold selection, if applicable: transforming continuous predictions into binary predictions: N/A | | |
|  | | **A**ssessment |  | | Performance statistics |  | | · Performance statistics estimated on training data: AUC, omission rates, MTSS | | |
|  |  |  |  | |  |  |  | · Performance statistics estimated on validation data (from data partitioning): AUC, omission rates, MTSS | | |
|  |  |  |  | |  |  |  | · Performance statistics estimated on test (truly independent) data: AUC, omission rates, MTSS | | |
|  |  |  |  | | Plausibility check |  | | · Response plots: independent response curves, jackknife test of variable importance | | |
|  |  |  |  |  |  |  | | · Expert judgements: Map display | | |
|  |  | **P**rediction |  |  | Prediction output |  |  | · Prediction unit: probability of presence [continuous] (%) | | |
|  |  |  |  |  |  |  | | · Post-processing, e.g. clipping, reprojection: N/A | | |
|  |  |  |  |  | Uncertainty quantification |  | | Uncertainty in scenarios (e.g. climate models, land use models, storylines):  · Bias in the climate model has been corrected using trend-preserving bias correction  · CHELSA climatic data were used, specifically CMIP6 ISIMP3, GFDL-ESM4, SSP 3-7.0. As all projected climate models will have uncertainty in them, additional information is available here: Brun P, Zimmermann NE, Hari C, Pellissier L, Karger DN. 2022a. Global climate-related predictors at kilometre resolution for the past and future. Earth Syst Sci Data Discuss, *14*(12), 5573-5603.[https://doi.org/10.5194/essd-2022-212]( https://doi.org/10.5194/essd-2022-212) | | |
|  |  |  |  |  |  |  | | · Visualisation/treatment of novel environments: Clamping | | |
